# Endorsement of Social and Personal Values Predicts the Desirability of Men and Women as Long-Term Partners

**DOI:** 10.1177/1474704917742384

**Published:** 2017-11-19

**Authors:** Guilherme S. Lopes, Nicole Barbaro, Yael Sela, Austin J. Jeffery, Michael N. Pham, Todd K. Shackelford, Virgil Zeigler-Hill

**Affiliations:** 1 Department of Psychology, Oakland University, Rochester, MI, USA

**Keywords:** human values, long-term mating, sex differences, partner preferences, evolutionary psychology

## Abstract

A prospective romantic partner’s desirability as a long-term partner may be affected by the values that he or she endorses. However, few studies have examined the effects of “values” on a person’s desirability as a long-term partner. We hypothesized that individuals who endorse social values (vs. personal values) will be perceived as more desirable long-term partners (Hypothesis 1) and that the endorsement of social values will be especially desirable in a male (vs. female) long-term partner (Hypothesis 2). The current study employed a 2 (sex of prospective partner: male vs. female) × 2 (values of prospective partner: personal vs. social) × 2 (physical attractiveness of prospective partner: unattractive vs. highly attractive) mixed-model design. Participants were 339 undergraduates (174 men, 165 women), with ages varying between 18 and 33 years (*M* = 19.9, *SD* = 3.6), and mostly in a romantic relationship (53.7%). Participants reported interest in a long-term relationship with prospective partners depicted in four scenarios (within subjects), each varying along the dimensions of values (personal vs. social) and physical attractiveness (unattractive vs. highly attractive). Individuals endorsing personal values (vs. social values) and men (vs. women) endorsing personal values were rated as less desirable as long-term partners. The current research adds to the partner preferences literature by demonstrating that an individual’s ascribed values influence others’ perceptions of desirability as a long-term partner and that these effects are consistently sex differentiated, as predicted by an evolutionary perspective on romantic partner preferences.

Long-term romantic relationships are the most common human mating arrangement (Buss, 2015). Over human evolutionary history, long-term relationships conferred benefits on both men and women. Men in long-term relationships may benefit from greater certainty of paternity, and women in long-term relationships may benefit from male-provisioned resources for her and her offspring (Gallup & Frederick, 2010). Men and women in long-term relationships also benefit from having a cooperative partner to help deal with survival challenges. A person’s psychological traits therefore may affect others’ perceptions of that person’s desirability as a long-term romantic partner including standing on personality dimensions (e.g., Lukaszewski & Roney, 2010), sense of humor (Miller, 2001), and willingness to invest in children (Brase, 2006; see Buss, 2015, for a review). One set of psychological factors that have been relatively neglected in the long-term partner preferences literature, however, is “values.” The current research aims to investigate whether a person’s endorsement of certain values may affect that person’s desirability as a long-term partner.

## Values

A value is a psychological feature that guides behaviors and cognitively represents needs (Gouveia, 2013). Values are correlated with other psychological constructs such as personality dimensions, interests, and attitudes (e.g., Olver & Mooradian, 2003), but values differ from these other constructs in important ways. For example, a personality dimension is a cluster of relatively fixed attributes, whereas values vary more throughout the life span (Rokeach, 1973). For example, neuroticism, extraversion, and openness are predominantly stable in adulthood (Costa et al., 1986), whereas endorsement of excitement values and existence values follows an inverted U-shaped pattern over the life span (Gouveia, Vione, Milfont, & Fischer, 2015). Values also differ from interests. An interest is focused on a specific object or situation (e.g., money; Rokeach, 1973), whereas a value represents needs that transcend specific objects or situations (e.g., wealth; Gouveia, 2013). Values are also not attitudes, because attitudes represent dispositions toward certain objects (e.g., art and people) or activities (e.g., occupations and hobbies; Rokeach, 1973), whereas values are cognitive representations of needs (e.g., freedom, knowledge, and prestige; Gouveia, 2013).

Schwartz’s typology of values has been widely used for empirical studies (see Schwartz, 2012). Over the years, however, this typology has specified multiple configurations of the value domain including 7 (Schwartz & Bilsky, 1990), 10 (Schwartz, 1992), 11 (Schwartz, 1994), and 19 (Schwartz, 2012) value types. These multiple configurations lack parsimony and theoretical focus, which may hinder scientific advancement when studies are to be compared or meta-analyzed (see Gouveia, Milfont, & Guerra, 2014a, 2014b; Schwartz, 2014). More recently, Gouveia proposed a more parsimonious, integrative theory of values, the functional theory of human values (Gouveia, 2003, 2013). Gouveia’s theory of values was based on previous values research and does not neglect or oppose existing models. It has generated empirical support from data collected from residents of all inhabited continents (for a review, see Gouveia, 2013).

Drawing on seminal work on values (e.g., Hofstede, 1984; Inglehart, 1977; Rokeach, 1973), Gouveia suggested a value typology that includes *personal values* and *social values*. Personal values facilitate the pursuit of personal goals (e.g., success, pleasure, and prestige; Gouveia, 2013), with people endorsing these values being more egocentric and intrapersonally focused. In contrast, social values facilitate interpersonal focus (e.g., social support, belongingness, and tradition), such that people endorsing these values prioritize social relationships. Although personal and social values are not in opposition to each other, they have the lowest correlation among values (i.e., they are relatively independent constructs; Gouveia, 2013). In the current research, we argue that personal values and social values may affect an individual’s desirability as a long-term partner.

## Values and Desirability as a Long-Term Partner

The adoption of certain values may be understood as solutions to adaptive problems of reproduction (Gouveia et al., 2014a). For example, particular values may serve as cues to infidelity risk. The endorsement of values such as pleasure and sexuality suggests that an individual may pursue immediate desire gratification (Gouveia et al., 2014a). The endorsement of such values may serve as a cue to others about the individual’s desire for novel sexual partners, which is potentially important because individuals who have had more sexual partners are more likely to be unfaithful than individuals who have had fewer sexual partners (Buss, 2015). In contrast, the endorsement of values such as tradition, belongingness, and obedience suggests greater interest in having a family and respecting cultural norms (Gouveia, 2013). Individuals who endorse such values display more loyalty and commitment in a romantic relationship (Guerra, Gouveia, Sousa, Lima, & Freires, 2012), which may be perceived by others as desirable traits for a long-term partner (Buss & Schmitt, 1993). An individual may therefore be more desirable as a long-term partner if he or she endorses values that signal greater commitment to romantic relationships.

Individuals endorsing personal values (e.g., pleasure, prestige, emotion, and success) report greater interest in changes in the social status quo and are interested in increasing the esteem that others have for them (e.g., Gouveia et al., 2014a). Such individuals are likely to seek their own personal benefits and to focus on securing social recognition and social status (Gouveia, 2013). For example, the endorsement of personal values is associated with narcissistic traits (e.g., self-promoting mate attraction behaviors are positively associated with Narcissism; Monteiro et al., 2017), suggesting that individuals endorsing personal values may be more likely to prioritize their own interests over the interests of others. Individuals endorsing personal values—such as pleasure and sexuality—are also more likely to engage in risk-taking activities including heavy alcohol consumption (Medeiros, Pimentel, Monteiro, Gouveia, & Medeiros, 2015) and infidelity (Guerra et al., 2012). Men engaging in risk-taking activities may be less desirable in a long-term mating context to the extent that risk-taking signals greater likelihood of infidelity (Buss & Schmitt, 1993). Additionally, although the endorsement of personal values may be attractive to women because it may cue men’s potential to acquire resources, individuals motivated by personal values are not necessarily willing to share resources, and prior studies indicate that features such as success and dominance are only desirable if accompanied by altruism (e.g., Jensen-Campbell, Graziano, & West, 1995). Therefore, endorsement of personal values may be perceived by men and women as an undesirable attribute of a long-term partner.

Individuals endorsing social values (e.g., affection, tradition, support, and obedience) express greater interest in social affiliation and interpersonal relationships, displaying more affective attributes such as expressions of love and care (Gouveia et al., 2014a). Such individuals are more likely to seek social stability and welfare (Gouveia et al., 2014a) and to pursue long-term relationships (Gouveia, 2013). Additionally, individuals endorsing social values emphasize the importance of social rules and norms, reflecting an interest in preserving cultural and conventional norms (Guerra et al., 2012). Such individuals are more likely to endorse sexual fidelity (Gouveia, 2013), which is particularly desired by men in a prospective long-term partner (Prokop, Obertová, & Fedor, 2010). Endorsement of social values is also associated with greater interest in provisioning resources to offspring, which is particularly desired by women in a prospective long-term partner (Schmitt et al., 2012). Moreover, the endorsement of social values is associated with agreeable characteristics such as kindness and empathy (Olver & Mooradian, 2003), which are weighted as the most important characteristics in a long-term partner for both men and women, cross-culturally (i.e., “kind and understanding”; Buss, 1989). Therefore, endorsement of social values may be perceived by men and women as a desirable attribute of a long-term partner.

Previous research has investigated the importance given to values, cross-culturally (e.g., Fischer, Milfont, & Gouveia, 2011; Schwartz & Bardi, 2001). However, no previous research has investigated the specific effects of endorsement of certain values on that individual’s desirability as a long-term partner. For example, Schwartz and Bardi (2001) found that benevolence values (conceptually equivalent to social values; see Gouveia et al., 2014a) are among the most important to individuals in many cultures, whereas power values (conceptually equivalent to personal values) are among the least important. However, Schwartz and Bardi (2001) investigated preferences for certain values, which is a broader assessment than desirability as a long-term partner. Lopes, Santos, Shackelford, Tratner, and Gouveia (2017) found that attractive men’s desirability as a long-term partner varies with men’s endorsement of excitement values. However, Lopes et al. considered a specific type of values (vs. a broader type, such as personal values) and did not compare the effects of personal values and social values on desirability as a long-term partner. In the current study, we address these gaps by investigating the effects of endorsement of personal values and social values on desirability as a long-term partner.

Endorsement of personal values indicates greater likelihood of engaging in risk-taking activities and, therefore, may serve as a cue of higher infidelity risk (compared to endorsement of social values). Endorsement of personal values may also suggest more self-promoting behaviors and less commitment to long-term relationships. In contrast, endorsement of social values indicates greater emphasis on the importance of social rules and norms and, therefore, may serve as a cue of lower infidelity risk (compared to endorsement of personal values). Endorsement of social values also suggests greater importance of having a family and stable interpersonal relationships. Because the endorsement of social values and personal values is generally associated with features that may be perceived as highly attractive and unattractive in a long-term context, respectively—for example, personal and social values may serve as cues to high and low infidelity risk, respectively—we hypothesized that prospective romantic partners who endorse social values (vs. personal values) will be perceived as more desirable long-term partners (Hypothesis 1). Additionally, given that social values are a desirable trait for a long-term partner and given that women (relative to men) are more selective about prospective long-term partners (Buss & Schmitt, 1993; Trivers, 1972), we hypothesized that women, more than men, will perceive prospective partners who endorse social values as a desirable long-term partner (Hypothesis 2).

The desirability of a prospective partner’s psychological traits may be weighted differently depending on that prospective partner’s physical attractiveness. For example, men are likely to perceive women as more desirable when they display cues to greater fertility (Schmitt et al., 2012), and cues to fertility are related to physical characteristics (Buss, 2015). Men more than women place a premium on physical attributes when considering prospective mating partners, so it is possible that certain psychological traits may not be powerful enough to have an impact on men’s evaluation of women who are physically unattractive. This suggests that the relationship between a prospective partner’s values and their desirability as a long-term partner may be qualified by the prospective partner’s physical attractiveness. We therefore controlled for prospective partner’s physical attractiveness in tests of the hypotheses. The current study employed a 2 (sex of prospective partner: male vs. female) × 2 (values of prospective partner: personal vs. social) × 2 (physical attractiveness of prospective partner: unattractive vs. highly attractive) mixed-model design.

## Method

### Participants and Procedure

Participants were 339 undergraduates (174 men, 165 women), with ages varying between 18 and 33 years (*M* = 19.9, *SD* = 3.6), and mostly in a romantic relationship (53.7%). Prospective participants from the Psychology Department Subject Pool at a large Midwestern University viewed an advertisement for the study on the university’s experiment management system. Interested and eligible participants were provided a link to the online study. Participants engaged in a romantic relationship were instructed to imagine that they were not in a relationship, so that they could more reliably assess an individual’s desirability as a long-term partner. Consenting participants completed an online survey and received partial course credit upon completion. Participants completed their participation in the study via a secure website.

Participants were presented with four scenarios that described a prospective opposite-sex romantic partner. Each scenario contained a unique combination of the two target variables: ascribed values (personal vs. social) and physical attractiveness (unattractive vs. highly attractive). Each participant was presented with each of the four scenarios (within participants) depicting an opposite-sex individual (between participants). For example, one scenario for female participants depicted a *highly attractive male* described as endorsing personal values (the complete scenarios are provided in the Appendix). We conducted a within-participants design (vs. between-participants design) for the scenarios because within-participants designs (vs. between-participants designs) facilitate the detection of smaller affects across the scenarios and require fewer participants to generate reliable data (Charness, Gneezy, & Kuhn, 2012). The ascribed value descriptions were adapted from the basic value survey (BVS; Gouveia, 2003) items’ content into a single, brief narrative. The personal values descriptions were derived from the BVS items referring to personal values (e.g., pleasure, prestige, and success), whereas the social values descriptions were derived from the BVS items referring to social values (e.g., affection and tradition obedience). An author of the BVS compared the contents of each narrative and their corresponding items, suggesting minor modifications to improve the narratives’ quality. This procedure resulted in two narratives that reliably represent personal or social values. The narratives are unique in their content and represent relatively independent constructs (i.e., personal values and social values; Gouveia, 2013). Each narrative had a female version and a male version, which were identical except for the individuals’ names (e.g., “Stephanie” and “Robert”). The narratives contained different names of each sex to prevent participant fatigue (e.g., the female version had Stephanie, “Claire,” “Sophia,” and “Mary”). The female version for social values was “She wants to have a deep and enduring affectionate relationship with someone with whom she can share successes and failures. She likes to feel that she is not alone in the world to form part of a social group and to have good neighborly relationships. She respects the traditions of the society she lives in, as well as her parents, superiors, and elders. She tries to follow the social norms and to fulfill her daily duties and obligations.” Narratives have been demonstrated to be adequate for use in value research (e.g., Lopes, Santos, Shackelford, Tratner, & Gouveia, 2017).

The prospective partner’s physical attractiveness was described by a score on a 10-point scale. *Highly attractive* prospective partners were described as a “9 or 10” on a 10-point scale, whereas *unattractive* prospective partners were described as a “4 or 5” on a 10-point scale. We chose values near the midpoint of the scale to represent unattractive targets to prevent floor effects (i.e., participants may not have been interested in a long-term relationship with an extremely unattractive person). We used descriptions (as opposed to displaying pictures) to control for potential confounds in the perception of attractiveness across the scenarios. This procedure has been used in previous research (e.g., Winegard, Winegard, Reynolds, Geary, & Baumeister, 2017). The presentation order of the scenarios was counterbalanced across participants. After reading each scenario, participants were asked to describe how interested they would be in having a long-term relationship with the person, using a scale that ranged from 1 (*not at all*) to 7 (*extremely*). Single-item assessments of physical attractiveness are adequate for use in partner preferences research (e.g., Edlund & Sagarin, 2014).

## Results

We conducted a three-way mixed-model analysis of variance (ANOVA) in which the *sex of the prospective partner* (male vs. female) was entered as a between-subjects factor, and the *ascribed values of the prospective partner* (personal vs. social) and the *physical attractiveness of the prospective partner* (unattractive vs. highly attractive) were entered as within-subjects factors. The desirability of the prospective partner as a long-term partner was the dependent variable. We probed significant interactions by using simple effects tests with a Bonferroni correction to avoid inflation of type I error rates.

**Figure 1.**
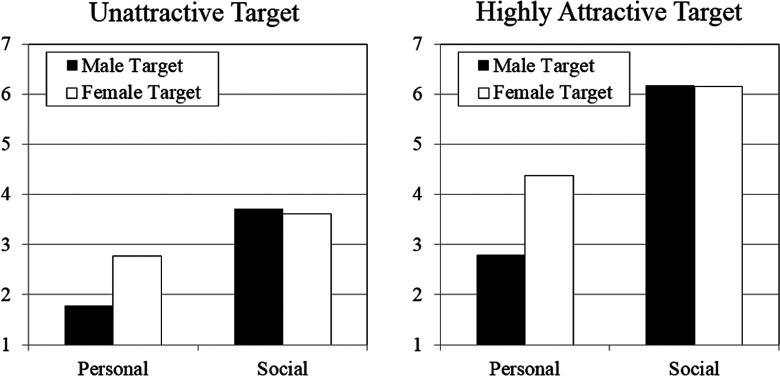
Effects of target’s sex, ascribed values, and physical attractiveness on their desirability as a long-term partner.

The results of the three-way mixed-model ANOVA indicated that long-term desirability varied according to prospective partner’s sex, *F*(1, 337) = 36.16, *p* < .001, η^2^ = .10; ascribed values, *F*(1, 337) = 499.44, *p* < .001, η^2^ = .60; and physical attractiveness, *F*(1, 337) = 644.07, *p* < .001, η^2^ = .66. These main effects were qualified by two-way and three-way interactions: Sex × Prospective Partner’s Physical Attractiveness, *F*(1, 337) = 5.10, *p* < .05, η^2^ = .02; Sex × Ascribed Values, *F*(1, 337) = 58.08, *p* < .001, η^2^ = .15; Ascribed Values × Prospective Partner’s Physical Attractiveness, *F*(1, 337) = 108.64, *p* < .001, η^2^ = .24; and the interaction between all three variables, *F*(1, 337) = 5.18, *p* < .05, η^2^ = .02; see Figure 1. Supporting Hypothesis 1, simple effects tests revealed that individuals ascribed with social values were rated as more desirable long-term partners than individuals ascribed with personal values, regardless of their physical attractiveness or sex. No sex differences were found for individuals ascribed with social values regardless of whether they were described as unattractive (*t* = 0.60, *p* = .548, *d* = .07) or highly attractive (*t* = 0.21, *p* = .833, *d* = .03), failing to support Hypothesis 2. Further, men were rated as less desirable long-term partners than women when they were ascribed with personal values. This pattern emerged regardless of whether the target was described as unattractive (*t* = 6.48, *p* < .001, *d* = .70) or highly attractive (*t* = 8.17, *p* < .001, *d* = .89). Results were unchanged when controlling for the participant’s current relationship status (analyses available on request).

## Discussion

The current study investigated the influence of an individual’s endorsement of certain values on their desirability as a long-term partner. We hypothesized that ascribed social values (more than personal values) of a target would increase his or her desirability as a long-term partner and that these effects would be sex differentiated. The results of the current study provided some support for this hypothesis in that desirability as a long-term partner depended on the sex of the target, the physical attractiveness of the target, and the values ascribed to the target. Specifically, individuals endorsing social values (vs. personal values) were rated as more desirable long-term partners, men (vs. women) endorsing social values were not rated as less desirable in a long-term context, and men (vs. women) endorsing personal values were rated as less desirable in a long-term context.

Endorsement of social values (e.g., affection and belonging) suggests greater interest in a long-term relationship (e.g., “He wants to have a deep and enduring affectionate relationship with someone with whom he can share successes and failures”; Gouveia, 2013). Additionally, people may perceive an individual’s interest in a long-term relationship as that individual’s willingness to provide resources for offspring—especially desirable to women in a long-term partner (Schmitt et al., 2012). Further, the description of social values depicts values such as “tradition” and “obedience” (e.g., “He tries to follow the social norms and to fulfill his daily duties and obligations”), suggesting a prosociality toward one’s social group, and the performance of prosocial, altruistic behaviors is positively associated with mating success (e.g., Arnocky, Piché, Albert, Ouellette, & Barclay, 2017). Individuals in a long-term relationship may reap several social benefits such as an increase in status and access to coalitional allies (Buss, 2015), ultimately increasing the health and well-being of their offspring. The endorsement of social values suggests a greater interest in establishing social networks that may lead to such coalitional allies (e.g., “He likes…to form part of a social group and to have good neighborly relationships”).

In contrast, endorsement of personal values suggests a focus on short-term achievements (e.g., “She likes to live for the moment and to satisfy all her desires”) and a greater interest in risk-taking activities (e.g., “She enjoys challenges and unknown situations and is always looking for risky adventures”). These inclinations may be perceived as undesirable in a long-term mating context because they signal greater likelihood of infidelity, and both men and women incur costs from a long-term partner’s infidelity. Men may unwittingly invest time and resources into genetically unrelated offspring at the expense of alternative mating opportunities (Buss & Schmitt, 1993), and a woman whose partner is unfaithful risks losing partner-provisioned resources should these be diverted to another woman (Schutzwohl & Koch, 2004). That is, psychosocial traits that signal a higher likelihood of infidelity decrease one’s desirability as a long-term partner.

Individuals endorsing personal values are more likely to display greater resource-acquisition potential, as suggested by values such as “prestige” (i.e., “He wants to receive respect for his contributions when he gets older”; Gouveia, 2013). However, a greater potential for acquiring resources does not necessarily indicate a willingness to share resources—for example, more masculine men are less willing to share resources with their long-term partner (Price, Kang, Dunn, & Hopkins, 2011). Additionally, the endorsement of personal values may signal sexual promiscuity (e.g., “She needs to have sex frequently to feel sexually satisfied”), which may be undesirable in a long-term context, as people who have had more (compared to fewer) sexual partners are more likely to be unfaithful in a long-term relationship (Buss, 2015). Moreover, personal values include values such as “power” (i.e., “He tries to…have the power to influence others and to control decisions”), which may be perceived as indicative of psychopathic personality traits (e.g., Patrick, Fowles, & Krueger, 2009) and undesirable characteristics in a long-term partner (e.g., psychopathy is associated with risky sexual behavior; Fulton, Marcus, & Zeigler-Hill, 2014).

Women prioritize psychological traits over physical traits in a long-term mating context (Williams, Fisher, & Cox, 2008), and therefore, women are expected to be more sensitive to psychological traits (relative to physical traits) than men in assessments of a prospective long-term partner’s desirability. This may explain why the magnitude of a value’s influence on long-term mating desirability was larger for female than for male participants. Endorsement of social values, on the other hand, was rated as equally desirable by female and male participants (see Figure 1), suggesting that the sexes similarly benefit from a long-term partner who endorses social values. A possible explanation is that endorsement of social values signals loyalty and commitment (e.g., “He wants to have a deep and enduring affectionate relationship”), suggesting greater certainty of paternity (for men) and partner’s investment in offspring (for women)—outcomes that are highly valued by men and women in a long-term mating context, respectively (Buss, 2015).

The results revealed an unpredicted sex differences in endorsement of personal values. Specifically, when ascribed with personal values, men were rated as less desirable than women, suggesting that the potential costs of having a long-term partner who endorses personal values are greater for women than for men. This result is consistent with previous research documenting that men’s (but not women’s) desirability as a long-term partner varies with ascribed excitement values (Lopes et al., 2017)—a class of personal values associated with risky activities (e.g., alcohol abuse; Medeiros et al., 2015) and infidelity (Guerra et al., 2012). These partner attributes are undesirable for both sexes in a long-term relationship, but because women prioritize psychological attributes over physical features in a long-term context (Williams et al., 2008), any psychological attribute that may lead to less parental investment—such as taking excessive risks that may reduce offspring survival, or committing emotional infidelity that may lead to diversion of resources—should be highly weighted in women’s evaluation of men’s desirability as a long-term partner.

This study offers several contributions to the partner preferences literature. First, it integrates a theory of values applicable in more than 50 countries (Gouveia et al., 2014a) with the metatheory of evolutionary psychology. We documented that ascribed values influence others’ perceptions of an individual’s desirability as a long-term partner and that these effects are consistently sex differentiated, as predicted by an evolutionary perspective on romantic partner preferences. In the future, researchers may profitably examine existing value theories with evolutionarily informed hypotheses of partner preferences, such as whether, which, and how values interact with mating criteria. The current results may also inform relationship counseling and therapy. For example, that men and women desire some values over others in a long-term romantic partner could be used in developing educational programs, marital counseling, and therapy plans.

The current study did not investigate the effects of personal values on social values (and vice versa). This design has the advantage of not prompting participants with statements that are not directly related to the type of values evaluated (i.e., personal values or social values), thus facilitating the detection of smaller affects for each value. However, future research may investigate the interaction between values on one’s desirability as a long-term partner such as the desirability as a long-term partner of individuals who endorse both personal and social values. Additionally, future research may benefit from investigating the mediational role of perceived risk of infidelity on the effects of a person’s endorsement of personal and social values on that person’s desirability as a long-term partner.

A limitation of the current study is that we did not assess participant’s own values. Controlling for participants’ values would allow researchers to test relevant value-specific hypotheses such as whether participants that endorse values similar to those ascribed to a prospective partner rate that potential partner as more desirable than a prospective partner ascribed with values that are different from those endorsed by the participant. Additionally, providing participants with information about a prospective partner’s values may trigger social stereotypes (e.g., “He likes to live for the moment and to satisfy all her desires” may trigger stereotypes of, e.g., *bon vivant*, which may bias some of the participants’ responses). Future research might investigate whether endorsement of certain values is associated with social stereotypes. The evolutionary psychological hypotheses of partner preferences have been subjected to intense empirical scrutiny in North America and Western Europe. We recommend this research be extended to include assessments of Asian and South American participants.

Few studies have examined the effects of values on a person’s desirability as a long-term partner (e.g., Lopes et al., 2017; Segal-Caspi, Roccas, & Sagiv, 2012). The current research adds to the partner preferences literature by documenting that ascribed values affect an individual’s desirability as a long-term partner. Specifically, the current study provides evidence that individuals endorsing personal values (vs. social values) and men (vs. women) endorsing personal values are less desirable in a long-term romantic context, in line with evolutionary psychological hypotheses of partner preferences.

## Appendix

### Female Target, Highly Attractive, With Ascribed Personal Values

Stephanie is about your age. She is highly physically attractive (people would probably rate her as 9 or 10 on a 10-point scale). She likes to live for the moment and to satisfy all her desires. She enjoys challenges and unknown situations and is always looking for risky adventures. She needs to have sex frequently to feel sexually satisfied. She tries to be efficient in everything she does and likes to have the power to influence others and to control decisions. She would be delighted if a lot of people admired her. She wants to receive respect for her contributions when she gets older.

### Female Target, Highly Attractive, With Ascribed Social Values

Claire is about your age. She is highly physically attractive (people would probably rate her as 9 or 10 on a 10-point scale). She wants to have a deep and enduring affectionate relationship with someone with whom she can share successes and failures. She likes to feel that she is not alone in the world to form part of a social group and to have good neighborly relationships. She respects the traditions of the society she lives in, as well as her parents, superiors, and elders. She tries to follow the social norms and to fulfill her daily duties and obligations.

### Female Target, Unattractive, With Ascribed Personal Values

Sophia is about your age. She is physically unattractive (people would probably rate her as 4 or 5 on a 10-point scale). She likes to live for the moment and to satisfy all her desires. She enjoys challenges and unknown situations and is always looking for risky adventures. She needs to have sex frequently to feel sexually satisfied. She tries to be efficient in everything she does and likes to have the power to influence others and to control decisions. She would be delighted if a lot of people admired her. She wants to receive respect for her contributions when she gets older.

### Female Target, Unattractive, With Ascribed Social Values

Mary is about your age. She is physically unattractive (people would probably rate her as 4 or 5 on a 10-point scale). She wants to have a deep and enduring affectionate relationship with someone with whom she can share successes and failures. She likes to feel that she is not alone in the world to form part of a social group and to have good neighborly relationships. She respects the traditions of the society she lives in, as well as her parents, superiors, and elders. She tries to follow the social norms and to fulfill her daily duties and obligations.

### Male Target, Highly Attractive, With Ascribed Personal Values

John is about your age. He is highly physically attractive (people would probably rate him as 9 or 10 on a 10-point scale). He likes to live for the moment and to satisfy all his desires. He enjoys challenges and unknown situations and is always looking for risky adventures. He needs to have sex frequently to feel sexually satisfied. He tries to be efficient in everything he does and likes to have the power to influence others and to control decisions. He would be delighted if a lot of people admired him. He wants to receive respect for his contributions when he gets older.

### Male Target, Highly Attractive, With Ascribed Social Values

Robert is about your age. He is highly physically attractive (people would probably rate him as 9 or 10 on a 10-point scale). He wants to have a deep and enduring affectionate relationship with someone with whom he can share successes and failures. He likes to feel that he is not alone in the world, to form part of a social group, and to have good neighborly relationships. He respects the traditions of the society he lives in, as well as his parents, superiors, and elders. He tries to follow the social norms and to fulfill his daily duties and obligations.

### Male Target, Unattractive, With Ascribed Personal Values

William is about your age. He is physically unattractive (people would probably rate him as 1 or 2 on a 10-point scale). He likes to live for the moment and to satisfy all his desires. He enjoys challenges and unknown situations and is always looking for risky adventures. He needs to have sex frequently to feel sexually satisfied. He tries to be efficient in everything he does and likes to have the power to influence others and to control decisions. He would be delighted if a lot of people admired him. He wants to receive respect for his contributions when he gets older.

### Male Target, Unattractive, With Ascribed Social Values

David is about your age. He is physically unattractive (people would probably rate him as 1 or 2 on a 10-point scale). He wants to have a deep and enduring affectionate relationship with someone with whom he can share successes and failures. He likes to feel that he is not alone in the world, to form part of a social group, and to have good neighborly relationships. He respects the traditions of the society he lives in, as well as his parents, superiors, and elders. He tries to follow the social norms and to fulfill his daily duties and obligations.
